# Pin1 Binding to Phosphorylated PSD-95 Regulates the Number of Functional Excitatory Synapses

**DOI:** 10.3389/fnmol.2020.00010

**Published:** 2020-03-13

**Authors:** Jary Y. Delgado, Duncan Nall, Paul R. Selvin

**Affiliations:** ^1^Department of Neurobiology, The University of Chicago, Chicago, IL, United States; ^2^Department of Physics and Center for the Physics of Living Cells, University of Illinois at Urbana–Champaign, Urbana, IL, United States

**Keywords:** post-synaptic density protein 95, proline-directed phosphorylation, palmitoylation, excitatory synaptic transmission, Pin1, *cis–trans* isomerization

## Abstract

The post-synaptic density protein 95 (PSD-95) plays a central role in excitatory synapse development and synaptic plasticity. Phosphorylation of the N-terminus of PSD-95 at threonine 19 (T19) and serine 25 (S25) decreases PSD-95 stability at synapses; however, a molecular mechanism linking PSD-95 phosphorylation to altered synaptic stability is lacking. Here, we show that phosphorylation of T19/S25 recruits the phosphorylation-dependent peptidyl-prolyl *cis–trans* isomerase (Pin1) and reduces the palmitoylation of Cysteine 3 and Cysteine 5 in PSD-95. This reduction in PSD-95 palmitoylation accounts for the observed loss in the number of dendritic PSD-95 clusters, the increased AMPAR mobility, and the decreased number of functional excitatory synapses. We find the effects of Pin1 overexpression were all rescued by manipulations aimed at increasing the levels of PSD-95 palmitoylation. Therefore, Pin1 is a key signaling molecule that regulates the stability of excitatory synapses and may participate in the destabilization of PSD-95 following the induction of synaptic plasticity.

## Introduction

The post-synaptic density (PSD) of excitatory synapses contains multiple scaffolding proteins, many of which belong to the membrane-associated guanylate kinase (MAGUK) family of scaffold proteins (Sheng and Hoogenraad, 2007). Of the MAGUKs, the post-synaptic density protein 95 (PSD-95) contributes between 300 and 400 copies to the PSD, making it one of the most abundant proteins at synapses (Chen et al., 2008). PSD-95 serves a diverse set of roles at excitatory synapses (Sheng and Hoogenraad, 2007).

As a scaffolding protein, it helps enrich synaptic ionotropic glutamate receptors of the α-amino-3-hydroxy-5-methyl-4-isoxazolepropionic acid (AMPA) and *N*-methyl-D-aspartate receptor (NMDAR) types in the post-synaptic membrane (Nair et al., 2013; Chen et al., 2015). The enrichment of AMPAR and NMDARs occurs via the interaction between the C-terminus PDZ binding motifs of the transmembrane AMPAR regulatory proteins (TARPS) or the C-terminus tail of the NMDAR-GluN2 subunits and one of the PDZ binding domains in PSD-95 (Sheng and Hoogenraad, 2007). In addition to its scaffolding function, PSD-95 is also involved in transsynaptic signaling (Mondin et al., 2011), regulation of presynaptic release (Futai et al., 2007), organization of signaling complexes downstream of NMDA receptors (Coba et al., 2009), and development of functional excitatory synapses (El-Husseini et al., 2000, 2002; Elias et al., 2006; Sheng and Hoogenraad, 2007; Chen et al., 2015).

Recently, PSD-95 has been implicated in the induction and expression of synaptic plasticity in pyramidal neurons (Béïque et al., 2006; Ehrlich et al., 2007; Carlisle et al., 2008; Xu et al., 2008; Sun and Turrigiano, 2011; Levy et al., 2015). For example, overexpression of PSD-95 in pyramidal CA1 neurons results in enhanced excitatory synaptic transmission, occlusion of pairing-induced long-term potentiation (LTP), and enhanced NMDAR-dependent long-term depression (LTD) (Stein et al., 2003; Ehrlich and Malinow, 2004). These findings suggest that PSD-95 is essential for bidirectional synaptic plasticity. For example, synapses with high amounts of PSD-95, i.e. during PSD-95 overexpression, prevents further accumulations of AMPARs following NMDAR-dependent LTP while at these synapses the induction of NMDAR-dependent LTD and removal of synaptic AMPARs is facilitated. On the other hand, removing or knocking down PSD-95 impairs the induction of NMDAR-dependent LTD (Migaud et al., 1998; Beique and Andrade, 2002; Ehrlich et al., 2007). However, the precise molecular mechanisms regulating PSD-95 stability at synapse are not fully understood.

Phosphorylation of the N-terminus domain of PSD-95 by proline-directed kinases is known to regulate PSD-95 synaptic stability (Morabito et al., 2004; Nelson et al., 2013). In particular, NMDAR-dependent LTD or application of Aβ peptides to hippocampal cultures increases threonine 19 (T19) and serine (S25) phosphorylation, and this phosphorylation event leads to the loss of PSD-95 from post-synaptic spines (Colledge et al., 2003; Morabito et al., 2004; Roselli et al., 2005; Bianchetta et al., 2011; Nelson et al., 2013). A potential molecular mechanism linking NMDAR-dependent LTD to phosphorylation of the N-terminus domain of PSD-95 and increase in intracellular Ca^2+^ influx implicates Ca^2+^/calmodulin (Ca^2+^/CaM) (Zhang et al., 2014; Chowdhury et al., 2018).

These newly described findings highlight the loss of PSD-95 following NMDAR activation; but they don’t relate to how constitutive phosphorylation of T19 and S25 regulates PSD-95 synaptic accumulation. Moreover, a great fraction of PSD-95 at the PSD is T19/S25 phosphorylated (Morabito et al., 2004), which highlights the importance of understanding the role of this molecular mechanism in the regulation of baseline excitatory synaptic transmission.

A potential regulator of PSD-95 synaptic stability during baseline conditions could be forged by the phosphorylation-specific peptidyl-prolyl *cis–trans* isomerase (Pin1). Pin1 is a small cytosolic and ubiquitously expressed peptidyl-prolyl isomerase, whose target recognition is independent of the increases in cytosolic Ca^2+^. Pin1 consists of two major domains: an N-terminal WW domain [containing two tryptophan (W) residues] and a C-terminal catalytically active peptidyl-prolyl isomerase (PPIase) domain (Yaffe et al., 1997; Lu et al., 2007). Via its N-terminus WW domain, Pin1 binds to substrates that are phosphorylated at serine/threonine-proline residues (Siepe and Jentsch, 2009; Moretto-Zita et al., 2010; Lonati et al., 2014). The enzymatic function of Pin1 is carried out via its C-terminal peptidyl-prolyl isomerase domain, which mediates the *cis–trans* peptidyl-prolyl isomerization of the phosphorylated serine/threonine-proline residues (Verdecia et al., 2000). In most targets, the *cis–trans* isomerization triggers a strong conformational change in the target protein and, in many cases, consequently restores biological function to its target (Lu et al., 2007).

This work tests the hypothesis that Pin1 binding via its WW domain to the phosphorylated T19/S25 in PSD-95 regulates PSD-95 accumulation at the PSD of hippocampal neurons. The association of Pin1 to these sites blocks palmitoylation of C3 and C5 in PSD-95. We find the reduction in PSD-95 palmitoylation correlates well with the decreased amounts of PSD-95 in post-synaptic dendrites, decreased number in post-synaptic spines, and reduced number of functional excitatory synapses. The remaining synapses remain functional with normal amounts of AMPARs and PSD-95 molecules. The decrease amounts of PSD-95 leads to a slight increase in the mobility of surface AMPARs. Lastly, the reduction in number of PSD-95 clusters is restored by manipulations that increased global palmitoylation. This supports the idea that the effects of Pin1 on synaptic PSD-95 clusters are due to a reduction in PSD-95 palmitoylation as opposed to the downregulation of some unknown protein. This data shows how phosphorylation of the N-terminal domain of PSD-95, from normal synaptic physiological processes, regulates the development and maintenance of functional excitatory synapses. These findings support the hypothesis that Pin1 is an important regulator of excitatory synapse function in the hippocampus.

## Materials and Methods

### Cloning and cDNA Plasmids

The plasmid encoding PSD-95:EGFP was a gift from S. Okabe (Tokyo University, Japan). The hPF11:EGF was used with permission from Dr. Masaki FUKATA. The triple T19A, S25A, and S35A (**N3A**-PSD-95) PSD-95:EGFP mutants was generated using site directed mutagenesis following the manufacturers recommendations (Agilent Technologies) and sequence verified. First the T19A and S25A double mutation was introduced using the following primers set: sense – GAAATACCGCTACCAAGATGAAGACGCGCCCCCTCTGGAACACGCGCCGGCCCACC TCCCCAACCAGGCCAATTC and antisense – GAATTGGCCTGGTTGGGGAGGTGGGC CGGCGCGTGTTCCAGAGGGGGCGCGTCTTCATCTTGGTAGCGGTATTTC. Then the S35A mutation was introduced using the following primers: sense – GGCCCACCTCCCCAACCAGGCCAATGC GCCCCCTGTGATTGTCAACACGGACAC and antisense – GTGTCCGTGTTGACAATCACAGG GGGCGCATTGGCCTGGTTGGGGAGGTGGGCC. The GST-Pin1 was obtained from addgene, plasmid ID# 19027 as described in Yaffe et al. (1997). Pin1 was cloned into the pIRES2EGFP vector (Clonetech) by PCR of the Pin1 coding sequence from the GST-Pin1 expression plasmid using the following PCR primer set: sense – TTAAAGCTAGCGAATTCGGCACGAGGGAAGAT GGC and antisense – CCTTAGAATTCTACTGTGTGACGGTGGCAG using Nhe1 and EcoR1. The K63A mutant was generated using the following primer set: sense –CGCACCTGCTGGTG GCGCACAGCCAGTCAC and antisense – TGACTGGCTGTGCGCCACCAGCAGGTGCG. The Pin1 R68A, 69A (RR,AA) pIRES2EGFP double mutant was introduced using following primer set: sense – GCACCTGCTGGTGAAGCACAGCCAGTCAGCGGCGCCCTCGTCCTGGCGGC AGGAGAAG and antisense – CTTCTCCTGCCGCCAGGACGAGGGCGCCGCTGACTGGCT GTGCTTCACCAGCAGGTGC. The GST-Pin1 WW fusion protein was generated by inserting a stop codon using the following primer set: sense – GCCCAGCGGCAACAGCAGCAGTGGTGGC TAAAACGGGCAGGGGGAGCCTGCCAGGG and antisense CCCTGGCAGGCTCCCCCTG CCCGTTTTAGCCACCACTGCTGCTGTTGCCGCTGGGC. The Pin1 C113S pIRES2EGFP was generated using the following primers: sense – CTGGCCTCACAGTTCAGCGACTCCAGCTCAGCCAAGGCCAGGGGAG and the antisense – CTCCCCTGGCCTTGGCTGAGCTGGAGTCGCTGAACTGTGAGGCCAG. The short hairpin against Pin1 were obtained from Transomics. The contained the following sequences; *Kd (1)* TGCTGTTGACAGTG AGCGCTCCTGCTACTGTCACACAGT ATAGTGAAGCCACAGATGTATACTGTGTGACAGTAGCAGGAATGCCTACTGCCTCGGA; *Kd (2)* TGCTGTTGACAGTGAGCGCTCACGGATTCAGG CATCCATATAGTGAAGCCACAGATGTATATGGATGCCTGAATCCGTGAATGCCTACTGCCT CGGA; *Kd (3)* GCTGTTGACAGTGAGCGCTCACGGATTCAGGCATCCATATAGTGAAGCCAC AGATGTATATGGATGCCTGAATCCGTGAATGCCTACTGCCTCGGA. The T19 PSD-95 peptide sequences was cloned into the EKAR construct using the following primer set: sense – GTGGTCGACGGTACCGCGGACCGGTTACCAAGATGAAGACACGCCCCCTCTGGAACACGC AAAGCTGTCATTCCAATTCCCGC and antisense – GCGGGAATTGGAATGACAGCTTTG CGTGTTCCAGAGGGGGCGTGTCTTCATCTTGGTAACCGGTCCGCGGTACCGTCGAC CAC. For the S25 PSD-95 peptide sequences the following primer set were used: sense – GTGGTCGACGGTACCGCGGACCGGTCCCCCTCTGGAACACAGCCCGGCCCACCTCCCCGCAAAGCTGTCATTCCAATTCCCGC and antisense – GCGGGAATTGGAATGACAGCTTT GCGGGGAGGTGGGCCGGGCTGTGTTCCAGAGGGGGACCGGTCCGCGGTACCGTCGA CCAC. Lastly, the S35 PSD-95 peptide sequences was introduced using the following primer set: sense –GTGGTCGACGGTACCGCGGACCGGTCCCAACCAGGCCAATTCTCCCCCTGTG ATTGTCGCAAAGCTGTCATTCCAATTCCCGC and antisense – CGGGAATTGGAATGACAGC TTTGCGACAATCACAGGGGGAGAATTGGCCTGGTTGGGACCGGTCCGCGGTACCGTCG ACCAC. The GCaMP6S have been described previously (Chen et al., 2013).

### Hippocampal Cultured Neurons

Preparation of cultured neurons was performed by plating neurons at a density of 100 to 200K per well in a 6 well plate. In brief, hippocampal neurons from E18 embryos of either sex were cultured on glass coverslips coated with Poly-lysine as in Borgdorff and Choquet (2002). Neurons were plated in Neurobasal supplemented with B27 and glutamine. The day after plating, neurons were treated with 1 μM Ara-C to stop glia and microglia proliferation. Feedings were done every 4 days using low cysteine containing media (Hogins et al., 2011). At day *in vitro* 8–10 neurons were transfected using Effectene or Lipofectamine 2000 following the manufacturer’s recommendation. Between 1 and 2 μg of the respective cDNA was used per well. Experiments were performed on neurons between 11 and 20 DIV.

### PC12 Stable Cell Lines

In brief, PC12 were cultured in NEM supplemented with 10% CS, 5% HS, and 1X PenStrep. Cells were eletroporated using a Lonza electroporator using the neuronal setting following manufacture’s recommendation for pulsing and cDNA concentrations. Two days post-transfections cells were started at 1 μg/mL Puromycin which was enough to kill most cells. Surviving cells were left to grow until visually identified clones emerged. Individual clones were picked and transferred into 6 well plates to grow to confluency. Feedings were done every 4 days.

### Whole Cell Electrophysiology

Individual coverslips were transferred one at a time to a submerged chamber mounted on a fixed-stage upright microscope. They were continuously perfused with oxygenated artificial CSF at 33°C flowing (ACSF) at a rate of 2–3 ml/min containing (in mM) 115 NaCl, 3 KCl, 1 NaH_2_PO_4_, 25 NaHCO_3_, 1 MgCl_2_, 2 CaCl_2_, 1 sodium pyruvate, and 10 dextrose. Individual cells were identified at 400X magnification using infrared DIC optics and an infrared-sensitive camera. EGFP expressing cells were identified by fluorescence through an FITC filter set. Whole-cell somatic recordings were obtained with pulled glass micropipettes (somatic 2.5–5 MΩ). Pipettes were coated with paraffin to reduce the pipette capacitance. Pipettes were filled with intracellular solution containing (in mM) 115 K-gluconate, 10 KCl, 1 HEPES, 10 Na_2_phosphocreatine, 4 MgATP, 0.3 NaGTP, and 0.2 EGTA adjusted with KOH to pH 7.3–7.35 and osmolarity was adjusted to 290 mOsm with K-Gluconate. Miniature excitatory post-synaptic potentials were isolated by adding 0.2 μM tetrodotoxin (TTX) and 100 μM bicuculline to the ACSF. Cells were voltage clamped at −70 mV and recordings were accepted if input resistances were > 100 MΩ, holding currents less than −100 pA and series resistances were < 20 MΩ. No adjustment of offset potential was performed. Cover slips were changed after 30 min in the recording chamber. Electrophysiological recordings were made using a Multiclamp 200B amplifier (Axon Instruments) or a HEKA EPC 10 amplifier (HEKA). Signals were filtered at 2 kHz and digitized at 10 kHz. All data analyses were performed using the MiniAnalysis software for automatic detection of events. Events were visually inspected for correct selection. Between 50 and 200 events were then used to extract peak measurements and events times. The amplitude and single event decay time constants were measured from each mEPSC. Average values are reported for each cell.

### PSD-95 Staining

In an alternative immunostaining experiments, 3–5 days post-transfection cells were fixed in 4% Paraformaldehyde at room temperature for 20 min. Cells were then rinsed three times with 1X PBS, then 5 min in 50 mM NH4Cl, and three more quick rinses in 1X PBS. Cells were permeabilized in 0.1% Tx-100 PBS for 5 min followed by three quick PBS rinses. Cells were incubated in freshly made 0.5% Sodium Borohydrate in PBS for 5 min. Cells were quickly rinse in PBS and incubated in 2 mL of 1% BSA in PBS for 45 min followed by incubation in 100 μL of anti-Pin1 (1:500) and anti-PSD-95 (1:500) for 1 h or overnight at 4°C (Table 1). Cells were rinsed 3X in PBS and the Alexa fluor 647 anti-mouse (1:500), and Alexa fluor 488 anti-rabbit applied at a dilution of 1:500 for 1 h in 1% BSA in PBS. Cells were rinsed 5X in PBS, post fixed in 4% PFA and mounted in slow fade mounting media (Life technologies).

**TABLE 1:** Antibody/drug table.

Antibody	Company	Catalog #	Concentration	Application
Anti-Pin1	Santa Cruz	SC-5340	1\500	Immunofluorescence
Anti-PSD-95	Pierce	MA1-046	1\500	Immunofluorescence

**Chemicals**	**Company**	**Catalog #**	**Concentration**	**Application**

Palmostatin B	Calbiochem	178501	1 μM	Cell culture
DMSO	SIGMA	D8418	0.01%	Cell culture

### Spinning Disk Confocal

Cells were imaged using 3-I Marianas live-cell dual-camera Yokogawa CSU-X spinning disk confocal. AxioObserver platform with DualCam and two Evolve EM-CCD cameras, CFP/YFP and R/G cubes using 100X/1.45 oil objective. The solid state 488, 561, and 640 lasers were used with fiber switcher to excite the corresponding fluorophores as needed. The objective was mounted onto a piezo MadCityLabs piezo Z insert which was used to collect Z-stacks or using either a DMI6000 Leica microscope (Leica Microsystems, Wetzlar, Germany) equipped with a confocal Scanner Unit CSU-X1 (Yokogawa Electric Corporation, Tokyo, Japan) using a 100X NA 1.4 oil objective (objective specs) and a QuantEM:512SC (Photometrics, Tucson, AZ, United States) or a Zeiss Axiovert 200M equipped with a confocal Scanner Unit CSU-X1 (Yokogawa Electric Corporation, Tokyo, Japan) using a EC Plan-Neofluar 100X/1.3NA Oil objective and a Photometric Cascade II was used to collect the fluorescence intensities. We used the 473, 532, 561, and 638 nm to excited the corresponding fluorophores as needed. The objective was mounted onto a piezo P721.LLQ [Physik Instrumente (PI), Karlsruhe, Germany] which was used to collect Z stacks.

### Single Particle Tracking

The FIONA experiments were performed with a Nikon Ti Eclipse microscope with a Nikon APO 100 X objective (N.A. 1.49). The microscope stabilizes the sample in z-axis with the Perfect Focus System. An Agilent laser system MLC400B with 4 fiber-coupled lasers (405, 488, 561, and 640 nm) was used for illumination. Elements software from Nikon was used for data acquisition. A back illuminated EMCCD (Andor DU897) was used for recording. For 3-D imaging, a cylindrical lens (CVI Melles Griot, SCX-25.4-5000.0-C-425-675) of 10 m focal length was inserted below the back aperture of the objective. A motorized stage from ASI with a Piezo top plate (ASI PZ-2000FT) was used for x-y-z position control. A quad-band dichroic (Chroma, ZT405-488-561-640RPC) was used and band-pass emission filter 525/50, 600/50, 710/40, 641/75 was used for fluorescence imaging. Primary hippocampal cultures, labeling and single particle tracking and analysis experiments were performed as previous described in great detail. In brief, on 12–13 days *in vitro* (DIV), neurons were co-transfected with control, Pin1 O.E. or KD plasmids, GluA2-AP (1 μg/coverslip), and BirA-ER (1 μg/coverslip) by using Lipofectamine 2000 transfection reagent as in Lee et al. (2017). At 24 ∼ 72 h after transfection, the coverslips were transferred to warm imaging buffer (HBSS supplemented with 10 mM Hepes, 1 mM MgCl_2_, 1.2 mM CaCl_2_, and 2 mM D-glucose) for 5 min incubation and mounted onto an imaging dish (Warner RC-40LP). Neurons were incubated in imaging buffer containing 60 pM Atto647N Stretptavidin (supplemented with 30 pM biotin to help prevent crosslinking) and casein (∼40 times dilution; stock solution purchased from Vector labs, SP-5020) for 5 min at 30°C and washed with 5 ml of imaging buffer. Finally, 1 mL of Hibernate E (Brain Bits, LLC) was added to the imaging dish that was subsequently mounted on the microscope.

After focusing the sample in bright field, the Perfect Focus System was activated to minimize the sample drift in z direction. The samples were then scanned in the GFP channel (488 excitation, 525/50 emission) to locate transfected cells. A fluorescent image of the cells was taken for reference. To track the SA labeled receptors, 640 nm laser was used for excitation in the hi-low-fluorescence mode with an appropriate band-pass filter for collecting the fluorescence.

For the tracking data, centroids of the all the SAs were localized in all the frames. A Matlab code was used to recover the trajectories of the SAs. In brief, the code finds locations of SAs in time t, and searches for nearby SA in time t + 1 as the next point on the trajectory. In the 3-D single particle tracking experiment, the maximum displacement of a SA in one time step is set to be 1 μm. The diffusion coefficients from the trajectories were calculated in Matlab by fitting the first 4 points of mean-square-displacement curve.

### Optical miniNMDARs

SlideBook software was used to control acquisition of the spinning disk hardware. Cover slips were individually mounted on the imaging chamber and provided with 2 mL of solution containing (in mM) 105 NaCl, 3 KCl, 1 NaH_2_PO_4_, 10 HEPES, 25 NaHCO_3_, 0 MgCl_2_, 2 CaCl_2_,1 sodium pyruvate, and 10 dextrose. AMPAR-mediated transmission was blocked with 10 μM CNQX and action potentials were blocked with 1 μM TTX. pH was regulated via a OKO full enclosure incubator providing moistened CO_2_. Individual cells expressing GCaMP6S and the shRNA (expressing turboRFP) were identified by fluorescence through an FITC filter set using a 40X/1.3 oil objective. Imaging was performed using the 100X/1.45 oil objective. Cells remained healthy for hours in this system as evidence of constant activity and a constant low level of GCaMP6S fluorescence. The time series collected for 3 min with an integration time of 200 ms per frame. Cells were excited with laser intensity of 0.74 mW. Single Ca^2+^ spine events were isolated using region measurement tool in FIJI and exported to MiniAnalysis for peak detection using pClamp10.

### Image Analysis

Cluster areas were determined from the thresholded EGFP signal by adjusting the filter setting on the thresholding function in FIJI to include exclusively dendritic spines. All areas where included in the measurements and analyzed regions were saved for *post hoc* verification. Threshold value was kept constant across conditions and was adjusted on a per week basis to accommodate good cluster separation in control cells. To control for week to week variability experiments are normalized on a per week basis and parameters kept constant across conditions. Analyze particles option in FIJI was used to extract features from the image.

### Experimental Design and Statistical Analysis

At least two coverslips/condition were used on each data set and a minimum of two coverslips per week. Each experiment was repeated 2–3 weeks. Each coverslip was randomly assigned the group before transfection. Data collection was interleaved and controlled for time and order effects. Coverslips which looked in poor health after transfection were discarded from analysis. Samples from all group were acquired on a weekly basis to reduce variability, otherwise no data was included in final analysis. We tested for outliers on a weekly basis and they were eliminated after testing all groups using Prism online calculator at a significance level of *p* < 0.05. Normality testing was performed on every group using D’Agostino and Pearson omnibus normality test. Between groups statistical significance was calculated accordingly for each distribution and experiment design. Data was normalized on a weekly basis to compensate for week to week variability. Numerical averages are presented as mean ± SEM or as box plots Statistical analyses were created using GraphPad Prism 5.0. GraphPad Prism 5.0 reports statistics as and quote: “For each pair of columns, Prism reports the *p*-value as > 0.05, < 0.05, < 0.01, or < 0.001. The calculation of the *p*-value takes into account the number of comparisons you are making. If the null hypothesis is true (all data are sampled from populations with identical distributions, so all differences between groups are due to random sampling), then there is a 5% chance that at least one of the post-tests will have *p* < 0.05. The 5% chance does not apply to each comparison but rather to the entire family of comparisons.” Exact *p*-values are reported when provided.

## Results

### Pin1 Regulates PSD-95 N-Terminus Palmitoylation

**FIGURE 1:**
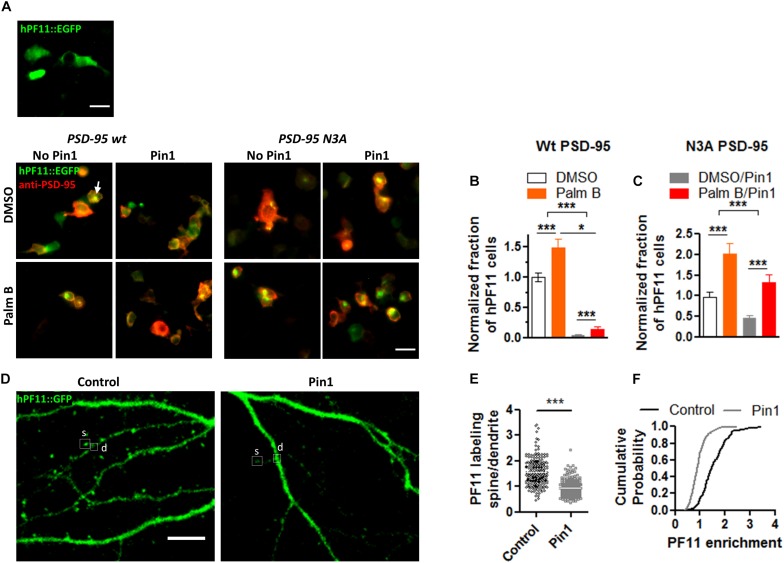
Pin1 reduces the amount of PSD-95 palmitoylation. **(A)** (top) Wide field images of HEK-293T cells transfected with only hPF11:EGFP. Notice the cytosolic non-punctate distribution. (bottom) DMSO or Palmostatin B-treated HEK 293T cells triple transfected with (1) hPF11:EGFP (green), (2) PSD-95 or the N3A mutant (red), and (3) empty or Pin1 expressing vectors. White arrows point to the intracellular clusters of PSD-95 as in Fukata et al. (2013) and Jeyifous et al. (2016). Scale bar: 10 μm. **(B)** Quantification of the effects of Pin1 overexpression on the formation of hPF11 intracellular clusters in DMSO and Palm B pretreatment in PSD-95 wt overexpressing cells. DMSO control 1.00 ± 0.07, *n* = 20; Palm B control 1.49 ± 0.14, *n* = 20; DMSO&Pin1 0.04 ± 0.01, *n* = 20; Palm B&Pin1 0.14 ± 0.05, *n* = 20. Two-way ANOVA *overexpression F*(1,76) = 13.43, *p* = 0.0005, *treatment F*(1,76) = 203.99, *p* < 0.0001, *interaction F*(1,76) = 5.84, *p* = 0.0181. **(C)** Quantification of the effects of Pin1 overexpression on the formation of hPF11 intracellular clusters in DMSO and Palm B pretreatment in N3A overexpressing cells. DMSO control 0.96 ± 0.12, *n* = 20; Palm B control 2.01 ± 0.26, *n* = 20; DMSO&Pin1 0.45 ± 0.07, *n* = 20; Palm B&Pin1 1.32 ± 0.19, *n* = 20. Two-way ANOVA *overexpression F*(1,76) = 30.78, *p* < 0.0001, treatment *F*(1,76) = 11.82, *p* = 0.0010, and the interaction *F*(1,76) = 0.28, *p* = 0.6015. **(D)** Confocal images of hippocampal cultured neurons at 18 days *in vitro* (DIV) expressing hPF11 in control and Pin1 overexpressing cells. The C113S isomerase mutant was used instead. Similar results were observed between the wt Pin1 and C113S. Scale bar: 10 μm. **(E)** Quantification of the enrichment ratio for hPF11 in control and Pin1 overexpressing neurons. Control 1.54 ± 0.04, 46 neurons, *n* = 213 spines; Pin1 0.93 ± 0.02, 49 neurons, *n* = 233 spines, Mann–Whitney test *p* < 0.0001. **(F)** Cumulative probability plot showing a reduction in the enrichment of hPF11. *p < 0.05 and ***p < 0.001.

We have shown that Pin1 can interact with the phosphorylated N-terminus domain of PSD-95 (in review). Furthermore, Ca^2+^/CaM association with these same residues in the N-terminus domain of PSD-95 blocks re-palmitoylation of cysteine 3 and 5 in PSD-95 (Chowdhury et al., 2018). Because palmitoylation of PSD-95 at C3 and C5 is necessary to stabilize PSD-95 within the post-synaptic densities (El-Husseini et al., 2002; Fukata et al., 2013; Park et al., 2013; Zhang et al., 2014) and Pin1 binds T19 and S25 in PSD-95 (under review), we tested if Pin1 can also regulate the levels of PSD-95 palmitoylation. PSD-95 palmitoylation was measured using an imaging approach utilizing a genetically encoded GFP fusion intrabody (hPF11) that specifically recognizes PSD-95 when it is palmitoylated (Fukata et al., 2013). Cells expressing only hPF11:EGFP don’t show clustering of the fluorescence and the fluorescence remains cytosolic (Figure 1A). Moreover, in heterologous cells expressing wt PSD-95, hPF11 recognizes palmitoylated PSD-95 localized to intracellular clusters that increase in size when global palmitoylation is increased (Jeyifous et al., 2016). Thus, hPF11 faithfully track decreases and increases in PSD-95 palmitoylation. To quantify the effects of Pin1 on PSD-95 palmitoylation, HEK cells were transfected with (1) either wt PSD-95 or N3A, the N-terminus mutant of PSD-95, (2) the hPF11 plasmid, and (3) either Pin1 or an empty vector (as control). The effects of Pin1 overexpression was quantified as the percentage of cells containing intracellular clusters (white arrows, Figure 1B). Also we tested the effects of increasing PSD-95 palmitoylation by treating cells with Palmostatin B (Palm B). We use a 6 h pretreatment with Palm B, an inhibitor of acyl protein thioesterase 1 that blocks depalmitoylation, which has been shown to increase the number of cells with visible hPF11 intracellular clusters over DMSO treated cells (Jeyifous et al., 2016). A two-way analysis of variance (ANOVA) design was used to test the interaction between Pin1-overexpressing cells and the increase in PSD-95 palmitoylation (Palm B). In DMSO-treated control there was a significantly higher number of hPF11 intracellular clusters than cells expressing Pin1 (Figure 1B). Similarly, a series of experiments aimed at confirming the relationships between palmitoylation measured using the hPF11 intrabody and the most stablished click chemistry assay (biochemically) show that with both methods Pin1 reduced the total level of PSD-95 palmitoylation down to 30% of control levels (e-mail communication with Antinone S. and Green N.W. Fri, Apr 24, 2015, 5:08 PM). Palm-B increased the number of PF11 clusters [Figure 1B, *F*(1,76) = 203.99, *p* < 0.0001]. The interaction between Pin1 overexpression and Palm B treatment was also significant, suggesting that Pin1 binding dominates over PSD-95 palmitoylation because Palm B failed to increase the number of hPF11 back to control levels, although a statistical significant increase in PSD-95 palmitoylation was observed. A two-way ANOVA experiment design was also used to test the interaction between Pin1 binding to the N-terminus mutant (**N3A**) of PSD-95 and its palmitoylation. As observed in DMSO-treated control cells, Pin1 overexpression significantly decreased the number of PF11 clusters in the N3A expressing cells (Figure 1C), suggesting that Pin1 regulates global protein palmitoylation as well as palmitoylation of PSD-95 when it binds to the N-terminus domain. The roles of Pin1 in regulating global palmitoylation have not been studied to this time. Palm B treatment significantly increased the number of PF11 clusters in cell expressing the N3A mutant (Figure 1C), thus making it insensitive to the overexpression of Pin1. Taken together, this data suggests that Pin1 binding to the N-terminus domain decreases the rates of PSD-95 palmitoylation under basal conditions and under conditions where the action of the acyl protein thioesterase 1 is blocked with Palm B pretreatment.

To quantify the effects of Pin1 on PSD-95 palmitoylation in neurons, the hPF11 experiment was repeated in cultured neurons transfected with hPF11 and wt Pin1 or an empty vector (as control). The effects of Pin1 on hPF11 immunodistribution were quantified as a change in the ratio of the hPF11:EGFP signal coming from consecutive spines to that of the adjacent dendrite (Figures 1D,E). The level of hPF11 overexpression was fairly high, and it appears as if not all the hPF11 molecules were bound to PSD-95. Therefore some of the hPF11 remained cytosolic, but even under those circumstances, we observe enrichment in dendritic spines. Similar to the effects observed for the palmitoylation-deficient mutant of PSD-95, Pin1 overexpression significantly reduced the enrichment of hPF11 in dendritic spines (Figure 1D). In Pin1 overexpressing neurons, hPF11 showed equal enrichment between dendrites and post-synaptic spines (Figure 1E). Furthermore, fewer than twenty percent of spines in neurons overexpressing Pin1 showed some hPF11 enrichment in dendritic spines, while about 90 percent of spines in control neurons showed enrichment of hPF11 to post-synaptic spines (Figure 1F). Knocking down Pin1 didn’t have a strong effect on the accumulation of PF11 in dendritic spines, but a slight trend toward larger PF11 clusters was observed (data not shown).

**FIGURE 2:**
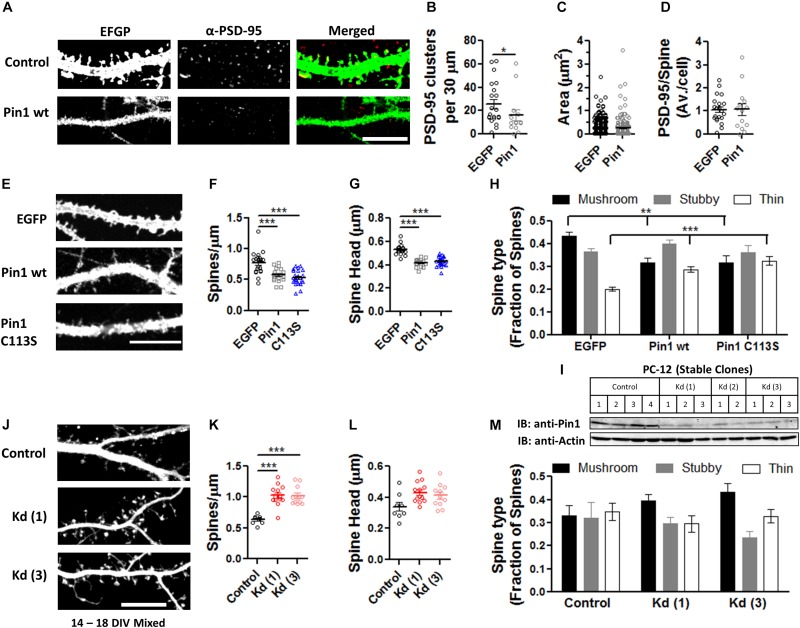
Pin1 regulates excitatory spines and PSD-95 protein levels. **(A)** Confocal images of hippocampal cultured neurons expressing EGFP and Pin1 IRES2 EGFP. The signal for EGFP, PSD-95, and overlay are shown. Middle panel shows the area analyzed. **(B)** Pin1 overexpression decreases the number of post-synaptic PSD-95 clusters. Number of clusters per 30 μm for EGFP 25.81 ± 4.06, *n* = 21; Pin1 15.79 ± 4.76, *n* = 14, Mann–Whitney test, *p* = 0.03. **(C)** Pin1 overexpression does not alter the size of post-synaptic PSD-95 punctate. EGFP 0.21 ± 0.010, *n* = 542; Pin1 0.24 ± 0.025, *n* = 221, Mann–Whitney test, *p* = 0.93. **(D)** Pin1 overexpression does not changes the number of the remaining spines with PSD-95 labeling. EGFP 1.06 ± 0.13; Pin1 1.07 ± 0.25, Mann–Whitney test, *p* = 0.66. For all scale bar 10 μm. **(E)** Confocal images of hippocampal cultured neurons at 14 days *in vitro* (range 12–14 DIV) expressing EGFP (control) or Pin1 or the C113S isomerase dead mutant. **(F)** Pin1 overexpression decreases the number of post-synaptic spines. EGFP 0.77 ± 0.04, *n* = 20; Pin1 0.57 ± 0.02, *n* = 20; C113S 0.53 ± 0.02, *n* = 25. One-way ANOVA *F*(2,62) = 18.98, *p* = 0.0001. **(G)** Pin1 overexpression reduces the size of post-synaptic dendritic spines. EGFP 0.53 ± 0.01, *n* = 20; Pin1 0.41 ± 0.01, *n* = 20; C113S 0.43 ± 0.01, *n* = 25, Kruskal–Wallis chi-square = 39.51, df = 2, *p* < 0.0001 **(H)** Pin1 overexpression alters the diversity of post-synaptic spines. *Control* mushroom 0.44 ± 0.01, stubby 0.36 ± 0.02, thin 0.20 ± 0.01, *n* = 20; *Pin1* mushroom 0.31 ± 0.02, stubby 0.40 ± 0.02, thin 0.29 ± 0.01, *n* = 20; C113S mushroom 0.32 ± 0.03, stubby 0.36 ± 0.03, thin 0.32 ± 0.02, *n* = 25. Two-way ANOVA *overexpression F*(2,186) < 0.001, *p* = 0.99, *spine type F*(2,186) = 18.33, *p* < 0.0001, the *interaction* was significant, *F*(4,186) = 9.242, *p* < 0.0001, **p = 0.013 **(I)** Western blots showing the level of knockdown of Pin1 protein from PC12 cell lysates stably expressing control shRNA or the various shRNA constructs (named Kd 1-3), a quantification of the knockdown of Pin1 from the western blot shown in (I), One-way ANOVA with Turkey’s multiple comparison test, *F*(3,8) = 23.06, *p* = 0.0003. **(J)** Confocal images of mixed-typed hippocampal cultured neurons at 18 days *in vitro* (range 14–18 DIV) expressing Turbo-mRFP with scramble shRNA or two shRNAs against the Pin1 mRNA. **(K)** Pin1 knockdown increases the number of post-synaptic spines. Control 0.63 ± 0.02, *n* = 8; Kd (1) 1.02 ± 0.05, *n* = 13; Kd (3) 1.02 ± 0.05, *n* = 11. Four coverslips/three different dissections. One-way ANOVA *F*(2, 29) = 22.32, *p* < 0.0001. **(L)** Pin1 knockdown slightly increases the size of post-synaptic dendritic spines. Control 0.34 ± 0.03, *n* = 8; Kd (1) 0.43 ± 0.02, *n* = 13; Kd (3) 0.41 ± 0.02, *n* = 11. One-way ANOVA *F*(2,29) = 3.995, *p* = 0.0293. **(M)** Pin1 knockdown triggers a slight increase in mushroom spines. control mushroom 0.33 ± 0.04, stubby 0.32 ± 0.05, thin 0.35 ± 0.04; Kd (1) mushroom 0.39 ± 0.03, stubby 0.30 ± 0.03, thin 0.3 ± 0.04; Kd (3) mushroom 0.43 ± 0.04, stubby 0.24 ± 0.03, thin 0.33 ± 0.03. Two-way ANOVA *overexpression F*(2,87) = 0.01, *p* = 0.99, *spine type F*(2,87) = 5.92, *p* = 0.0039, the *interaction*, *F*(4,87) = 1.93, *p* = 0.1124. ***p ≤ 0.001.

The loss of PSD-95 palmitoylation could lead to the loss of PSD-95 from dendritic spines and this loss could lead to a reduction in post-synaptic spine maturation (El-Husseini et al., 2000, 2002). To examine if Pin1 regulates the amounts of PSD-95 within post-synaptic spines and the number of post-synaptic PSD-95 clusters, cultured hippocampal neurons were transfected with pIRES2:EGFP or Pin1:IRES2:EGFP (expressing EGFP out of an Internal Ribosomal Entry Site, IRES) and immunostained for endogenous PSD-95 (Figure 2A). EGFP is used as a cytosolic marker. Strongly transfected neurons were not included in analysis. Cells overexpressing Pin1 showed a reduced number of PSD-95-positive clusters per 30 μm of dendritic section (Figure 2B) but did not altered the area or the number of PSD-95 clusters on the surviving spines (Figures 2C,D, respectively). This data suggests that Pin1 regulates PSD-95 palmitoylation, and this association limits the amount of PSD-95 that can become part of a post-synaptic cluster and as a result fewer clusters are formed. Once the PSD-95 clusters are formed, however, they are indistinguishable from the PSD-95 clusters present in control cells.

### Pin1 Regulates Post-synaptic Spines

The decrease in PSD-95 palmitoylation and number of PSD-95 clusters suggests that Pin1 could regulate dendritic spines because PSD-95 palmitoylation strongly regulates dendritic spine formation (El-Husseini et al., 2000, 2002). Furthermore, conflicting results are observed as to the role of Pin1 in the regulation of post-synaptic spines between the global KO (Antonelli et al., 2016) and the conditional KO (Stallings et al., 2018). For instance, the global Pin1 knockout mice show an increase in spine density (Antonelli et al., 2016) while a recent paper shows that deleting Pin1 from the adult hippocampus decreases it Stallings et al. (2018). These discrepancies in results, led us to reevaluate the role for post-synaptic Pin1 in dendritic spine morphology.

To calrify this controversy, the features of dendritic spines were thus analyzed in neurons overexpressing EGFP, Pin1, or Pin1 C113S (Figure 2E). The C113S mutation on the isomerase domain of Pin1 devoid the protein from its catalytic activity and this mutant form of Pin1 is not transported to the nucleus (Lufei and Cao, 2009). These two features allow us to dissociate the role of cytosolic Pin1 binding from isomerization as well as exclude any potential nuclear effects of Pin1. The number of post-synaptic spines per micron of dendrite was strongly decreased in neurons overexpressing Pin1 or the isomerase mutant C113S, suggesting that binding and not isomerization dominates this effect (Figure 2F). The width of post-synaptic spine heads was significantly reduced (Figure 2G). To analyze if Pin1 overexpression alters the diversity of post-synaptic spines, a factorial ANOVA was conducted to compare the main effects of overexpressed protein and the interaction effect between overexpression and the fraction of spines (spine type). The overexpression included three levels of analysis (EGFP, Pin1, and C113S) and the spine type also included three levels (mushroom, stubby, and thin). A 2-way ANOVA revealed that the overexpression was not statistically significant across the groups but the interaction (between the overexpressed protein and the type of spine) was significant. A Bonferroni post-test revealed that the diversity of spines is lost in Pin1 and C113S overexpressing neurons (Figure 2H). Similar to the results in the global Pin1 KO, knocking down Pin1 with an shRNA strategy increased the number of post-synaptic spines per micron of dendrite (Figures 2H–K). The Pin1 shRNA knocked down Pin1 down to 30% of control levels (Figure 2I). As for the overexpression experiment, a slight change toward post-synaptic spines with larger head-width was observed (Figures 2J,L), and most evident in the cumulative distribution curves (data not shown). To analyze if Pin1 knockdown altered the diversity of post-synaptic spines, a factorial ANOVA was conducted to compare the main effects of overexpressed protein and the interaction effect between overexpression and the fraction of spines (spine type). The overexpression included three levels [Control, Kd (1), and Kd (3)] and the spine type included three levels (mushroom, stubby, and thin). The overexpression was not statistically significant and no interaction was observed. Only a trend toward more mushroom spines was observed in cells expressing the knockdown plasmids (Figure 2M). Taken together, the overexpression and knockdown data indicates that post-synaptic Pin1 is a strong negative regulator of the number of post-synaptic spines.

### Post-synaptic Pin1 Regulates the Number of Functional Excitatory Synapses

**FIGURE 3:**
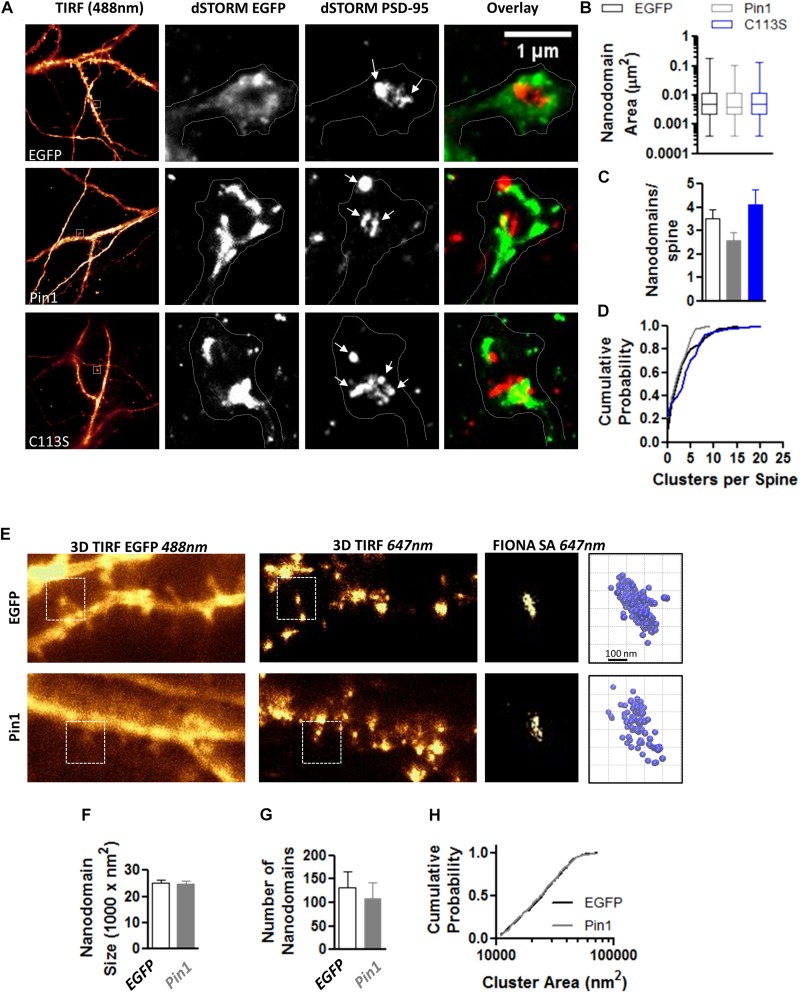
Pin1 does not regulate PSD-95 and AMPAR nanodomains. **(A)** Images of hippocampal cultured neurons at 16 DIV imaged under widefield TIRF illumination and the superresolution reconstruction of the 568 nm (for EGFP) and 647 nm (for PSD-95) channel. Overlay of dSTROM images for EGFP and PSD-95 shown on right. **(B)** Box plot graph showing similar sized nanodomains for neurons expressing EGFP, Pin1 or the C113S mutant. EGFP 0.013 ± 0.001, Pin1 0.012 ± 0.002, and C113S 0.011 ± 0.001 μm^2^, Kruskal–Wallis test with Dunn’s multiple comparison test, *p* = 0.35. **(C)** The average number of nanodomains per dendritic spine for neurons expressing EGFP, Pin1 or the C113S mutant. EGFP 3.52 ± 0.39, Pin1 2.58 ± 0.36, and C113S 4.12 ± 0.64, Kruskal–Wallis test with Dunn’s multiple comparison test, *p* = 0.31. **(D)** Cumulative probability plot of data shown on C showing a slight shift toward spines with a higher number of nanodomains for cells expressing the C113S. Unpaired *t*-test Pin1 vs. C113S, *p* = 0.02. **(E)** Live cell widefield TIRF images for EGFP channel and Streptavidin 647 nm stained GluA2 receptors (FIONA) for hippocampal cultured neurons transfected with EGFP or Pin1. Boxed regions show a high-resolution image for the GluA2 staining and 3D VMD plots of AMPAR nanodomains. **(F)** Pin1 overexpression does not alter the size of nanodomains. EGFP 25094 ± 1112, *n* = 8 cells, and Pin1 24993 ± 1025 nm^2^, *n* = 6 cells. Unpaired *t*-test *p* = 0.95. **(G)** Pin1 overexpression does not alter the number of nanodomains along dendritic fields. For EGFP cells 132.3 ± 34.09 nanodomains, *n* = 8 cells, and Pin1 109.2 ± 33.33, *n* = 6 cells. Unpaired *t*-test, *p* = 0.95. **(H)** Cumulative probability plot of data shown on G for EGFP and Pin1 expressing cells.

The reduction in the levels of PSD-95 palmitoylation and the loss of post-synaptic spines and PSD-95 clusters suggested that Pin1 could regulate many aspects of excitatory synaptic transmission. To evaluate if Pin1 affects the nano-organization of PSD-95 in post-synaptic spines, we performed super-resolution microscopy of endogenous PSD-95 in cultured neurons transfected with EGFP, Pin1 or the C113S mutant (Figure 3A). Previous studies have shown that AMPARs and PSD-95 lie in nanodomains, small clusters of about 70 nm in diameter (Nair et al., 2013; Cai et al., 2014; Constals et al., 2015; Li et al., 2016; Lee et al., 2017). It is hypothesized that the nanodomain organization of synaptic proteins plays a key role in excitatory synaptic transmission. We found that Pin1 overexpression did not affect the size of the PSD-95 nanodomains (Figure 3B) or the number of post-synaptic PSD-95 nanodomains per spine (Figures 3C,D). We also measured the size and number of AMPAR nanodomains because it has been hypothesized that these are the minimal unit of AMPAR-mediated transmission (Nair et al., 2013). We performed FIONA on neurons expressing EGFP or Pin1 and EGFP (Figure 3E) as in Yildiz et al. (2003), Cai et al. (2014), Lee et al. (2017). Pin1 overexpression did not alter the size of GluA2 nanodomains, the area, or the number of nanodomains (Figures 3F–H).

**FIGURE 4:**
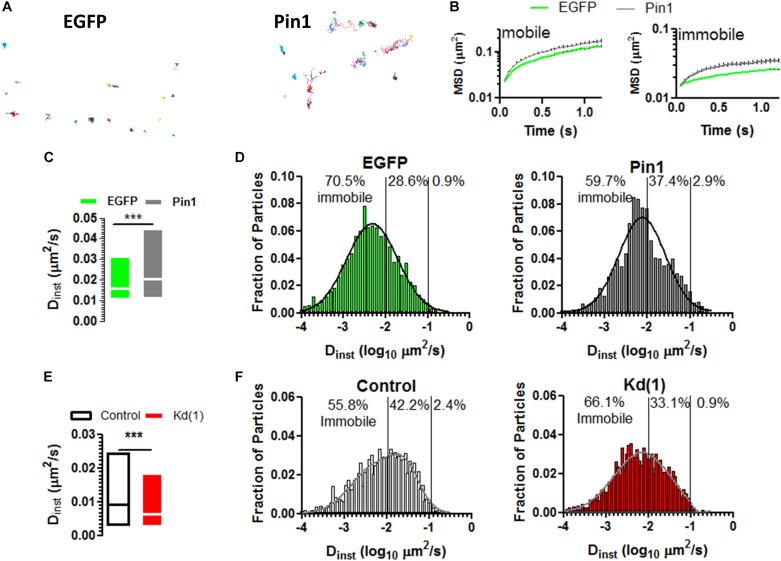
Pin1 weakly regulates the mobility of surface AMPARs. **(A)** Single particle tracks for neurons expressing EGFP or Pin1. **(B)** Representative plots of mean squared displacement for mobile particles with instantaneous diffusion coefficient (D_inst_) lower than 0.0073 μm^2^/s and immobile particles with lower mobility. **(C)** Box plots showing the D_inst_ for surface containing GluA2 receptors expressed in cells transfected with EGFP or Pin1. cells. EGFP 0.026 ± 0.0006 *n* = 3,473, 18 cells vs. Pin1 0.036 ± 0.0008 μm^2^/s *n* = 3,367, 18 cells Mann–Whitney test, *p* < 0.0001. **(D)** Histogram showing the fraction of particles plotted against their D_ints_ for EGFP (green) or Pin1 (dark gray) expressing cells. **(E)** Box plots showing the D_inst_ for surface containing GluA2 receptors expressed in cells transfected with Control or Kd (1) shRNA. Median Control 0.009 *n* = 3,576, 11 cells vs. Pin1 0.006 μm^2^/s *n* =, 12 cells Mann–Whitney test, *p* < 0.0001. **(F)** Histogram showing the fraction of particles plotted against their D_ints_ for Control (gray) or Kd (1) (red) expressing cells.

Given that Pin1 reduced the amounts of PSD-95 and on the remaining spines the amounts of PSD-95 and AMPARs remained unaltered, we reason that maybe Pin1 changed the mobility of AMPARs. The mobility of AMPARs is regulated by the induction of LTP and by the amounts of PSD-95 (Makino and Malinow, 2009; Czöndör et al., 2012), thus we reason that Pin1 may change the mobility of surface expressed AMPARs. To evaluate if Pin1 could affect the dynamics of the AMPAR we performed single particle tracking experiments on neurons transfected with (1) a biotinylated form of GluA2 subunit, (2) the biotin ligase BirA expressing plasmid and (3) either pIRES2:EGFP or Pin1:IRES2:EGFP (Figure 4A). We observed that Pin1 overexpression increased the mobility of slowly moving particles with instantaneous diffusion coefficient (D_inst_) lower than 0.007 μm^2^/s (Figures 4B–D, Mann–Whitney test, *p* < 0.0001) while knocking down Pin1 had the converse effect of slowing down the mobility of AMPARs (Figures 4E,F, Mann–Whitney test, *p* < 0.0001). Although, EGFP and mRFP turbo differ somewhat in the mean D_inst_, we tried to control to the best of our capacities, on a weekly basis, all factors known to regulate AMPAR mobility (i.e. developmental age, effects of transfection of culture viability, labeling density, etc.). Thus, Pin1 mildly affects the residence time of GluA2-containing AMPARs at synapses.

**FIGURE 5:**
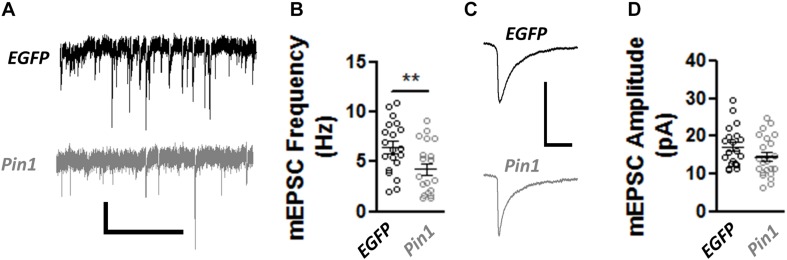
Pin1 regulates excitatory synaptic transmission. **(A)** Electrophysiological mEPSCs voltage clamp recordings from hippocampal cultured neurons transfected with EGFP and Pin1. Scale bar 20 pA/2sec. **(B)** Pin1 decreases the frequency of mEPSCs. Normalized frequency of mEPSCs. EGFP 6.45 ± 0.59, *n* = 20; Pin1 4.23 ± 0.58, *n* = 20, unpaired *t*-test, **p = 0.0109. **(C)** Average mEPSC traces. Scale bar 20 pA/20 ms. **(D)** Pin1 does not alter the amplitude of mEPSCs. EGFP 17.19 ± 1.19; Pin1 14.28 ± 1.27, *n* = 20, unpaired *t*-test, *p* = 0.11. Two dissociations, two recording sessions, and nine coverslips.

The decrease in post-synaptic spines, PSD-95 amounts, and the change in AMPAR mobility coupled with the normal size of GluA2 nanodomains in spines and dendrites suggests that Pin1 could regulate the number of functional excitatory synapses but not the strength of the remaining ones. To test this hypothesis, AMPA- and NMDA-receptor-mediated transmission was measured. The AMPAR-mediated transmission was recorded by isolating miniature EPSCs (mEPSCs) from cultured neurons transfected with EGFP or wt Pin1. As expected from the loss of post-synaptic spines, Pin1 overexpression strongly decreased the frequency of mEPSCs (Figures 5A,B) but did not alter the amplitude of the mEPSCs (Figures 5C,D).

**FIGURE 6:**
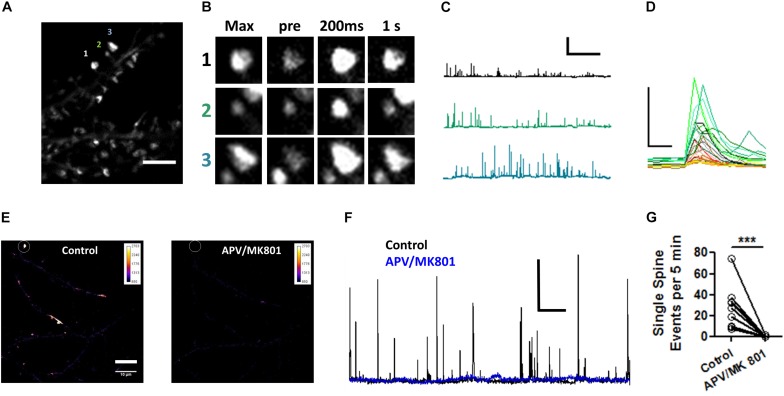
Visualization of NMDAR-mediated GCaMP6S Ca^2+^ signals in single spines. **(A)** Maximum projection of 200 s recordings at low laser intensities. Scale bar: 5 μm. **(B)** Regions of interest shown on **(A)**. Max projection zooms for regions 1–3. *pre* indicates the time point (–200 ms) immediately precedent an event, *200 ms* first time point in the event series, and *1 s* spine signal 1 s after the peak response. **(C)** Three one 500 s long traces showing the transient increases in fluorescence plotted as ΔF/F for the spines indicated in **(A)**. **(D)** Selected zoomed events from those shown on **(C)**, only 3 s are shown. Scale bar 5X ΔF/F and 500 ms. **(E)** Pseudocolor whole field view (100X) of maximum projection of dendrites expressing GCaMP6S for periods before and after 50 μM APV/10 μM MK801. **(F)** 1,500 s long traces showing the transient increases in fluorescence plotted as ΔF/F for the spines indicated in **(E)** for periods before and after APV/MK801 application.**(G)** Number of optical NMDAR minis before and after APV/MK801. *n* = 4 cells, 12 spines. Wilcoxon matched-pairs signed rank test, ***p = 0.0025.

**FIGURE 7:**
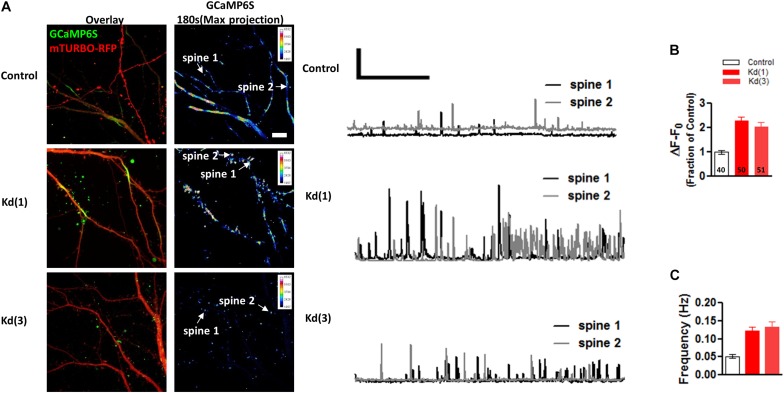
Pin1 downregulation increases the NMDAR-mediated GCaMP6S Ca^2+^ signals in single spines. **(A)** Spinning disk confocal images showing maximum projections of the GCaMP6S, Turbo-mRFP (shRNA channel), and the overlay image. Control and the two knockdown conditions are shown. 3 min of imaging time compressed into a maximum projection pseudocolor images of the GCaMP6S channel. Intensity scale shown on upper right quadrant. (middle) Two 180 s long traces showing the transient increases in fluorescence plotted as ΔF/F for the spines indicated in **(A)**. Scale bar 1X ΔF/F and 50 ms. **(B)** Quantification of ΔF/F signal intensity for control or knockdown expressing cells. Control 1.00 ± 0.09, n = 40 spines, 9 cells; Kd (1) 2.35 ± 0.17, *n* = 50, 14 cells; Kd (3) 2.19 ± 0.28, *n* = 51, 12 cells. One-way ANOVA with Bonferroni’s multiple comparison test, *F*(2,64) = 17.96, *p* < 0.0001. Scale bar: 10 μm. **(C)** Quantification of single spine event frequency for control or knockdown expressing cells. Control 0.05 ± 0.005, *n* = 40 spines, 9 cells; Kd (1) 0.13 ± 0.02, *n* = 50, 14 cells; Kd (3) 0.05 ± 0.01, *n* = 51, 12 cells. One-way ANOVA with Bonferroni’s multiple comparison test, *F*(2,64) = 8.81, *p* < 0.0004.

To confirm the effects of Pin1 on NMDAR currents as in Antonelli et al. (2016), NMDAR-mediated transmission was evaluated on by isolating single spine miniature NMDAR (mNMDARs) events by using a modified ACSF containing 0 mM Mg^2+^, 1 μM TTX, and 10 μM DNQX. Ca^2+^ entry through the NMDAR was imaged using the high-affinity Ca^2+^ sensor GCaMP6S in a spinning disk microscopy (Chen et al., 2013) (see Materials and Methods” and Supplementary Movie I). Ca^2+^ signals were detected in isolated dendritic spines (regions 1, 2, and 3 Figure 6A) and plotted as in Reese and Kavalali (2016), were characterized by a rapid increase in intracellular Ca^2+^ (Figure 6C and single events Figure 6D), and were blocked by APV and MK801 (Figures 6E–G). To test if Pin1 regulates these single spine mNMDARs in neurons, two of the three shRNA constructs were transfected into neurons. Knockdown Pin1 doubled the amplitude of mNMDARs (Figures 7A,B) as well as doubled the frequency of single spine mNMDARs (Figure 7C). No measurements on dendritic Ca^2+^ release were done. The increase in size and frequency of the mNMDARs supports the idea that post-synaptic Pin1 negatively regulates the amount of NMDARs either via binding to the N-terminus domain or plausibly via the hinge domain as implied by Antonelli et al. (2016). Furthermore, this data supports the hypothesis that Pin1 regulates the number of functional excitatory synapses in hippocampal neurons via its association with PSD-95.

### Overexpressing PSD-95 Overcomes the Effects of Overexpressing Pin1

**FIGURE 8:**
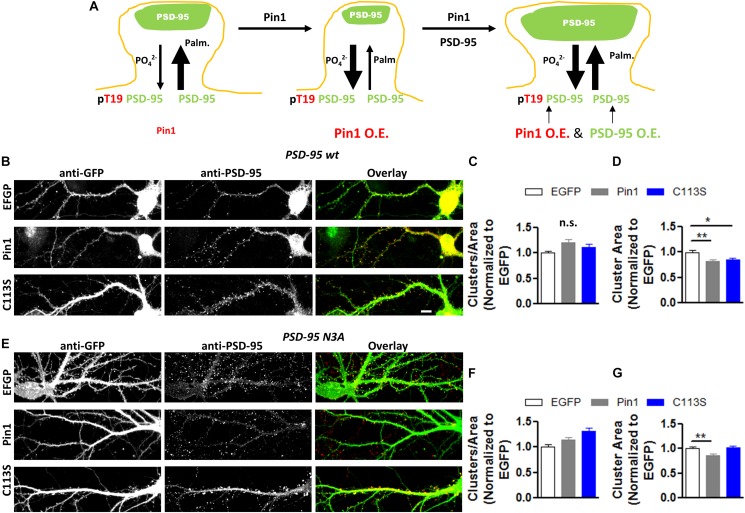
PSD-95 restores the decrease in PSD-95 cluster number induced by Pin1. **(A)** Model showing how post-translational modification regulates PSD-95 entry and stability at the PSD. **(B)** Confocal images of hippocampal cultured neurons overexpressing PSD-95 and control or Pin1 IRES EGFP. Images show EGFP, PSD-95 staining and the overlay image. Scale bar: 10 μm. **(C)** wt PSD-95 restores the decrease in cluster number. Normalized number of clusters for dendrites expressing EGFP EGFP 1.00 ± 0.04, *n* = 49; Pin1 1.21 ± 0.07, *n* = 53; C113S 1.12 ± 0.07, *n* = 54, **(D)** PSD-95 overexpression restores the area of PSD-95 clusters well above the level of non-transfected. EGFP 1.00 ± 0.04, *n* = 49; Pin1 0.82 ± 0.04, *n* = 53, C113S 0.85 ± 0.03, *n* = 55 Kruskal–Wallis chi-square = 13.46, df = 2, *p* < 0.0012. **(E)** Similar to wt PSD-95, overexpression of the N3A mutant of PSD-95 also restores to control levels the density of PSD-95 per dendritic area in cells overexpressing Pin1. **(F)** N3A overexpression restores the cluster of PSD-95 per dendritic area above the level of EGFP expressing cells. EGFP 1.00 ± 0.04, *n* = 42; Pin1 0.14 ± 0.05, *n* = 66; C113S 1.32 ± 0.06, *n* = 51, One-way ANOVA *F*(2,156) = 7.84, *p* = 0.0006. **(G)** N3A overexpression restores the area of PSD-95 clusters well above the level of non-transfected. EGFP 1.00 ± 0.03, *n* = 42; Pin1 0.86 ± 0.03, *n* = 66; C113S 1.02 ± 0.04, *n* = 51 55 Kruskal–Wallis chi-square = 16.21, df = 2, *p* < 0.0003.

The previous results support the hypothesis that Pin1 affects dendritic spines by interfering with the palmitoylation of PSD-95 and the development or maturation of post-synaptic spines. If the decreased number of post-synaptic spines and PSD-95 clusters are due to Pin1’s interference with PSD-95 palmitoylation, overexpression of PSD-95 should rescue the morphological effects as more of the PSD-95, from the total pool, will be palmitoylated PSD-95 (Figure 8A scheme). To test this hypothesis, PSD-95 was overexpressed with EGFP, Pin1 or the isomerization mutant C113S. Cultured hippocampal neurons were immunostained for PSD-95 (Figure 8B). PSD-95 overexpression restored the density of PSD-95 per dendritic area in cells overexpressing Pin1 (Figure 8C) previously observed in Figure 8B. In this experiment, we were unable to accurately quantify the effects of Pin1 overexpression on endogenous PSD-95 due to the strong labeling intensity of the cells overexpressing PSD-95. PSD-95 overexpression also restored the area of PSD-95 clusters well above the level of non-transfected neurons (which are dimly visible in the image); however, Pin1 binding still exerted a limiting effect on the size of PSD-95 puncta (Figure 8D). Similar to wt PSD-95, overexpression of the N3A mutant of PSD-95 also restored the density of PSD-95 per dendritic area in cells overexpressing Pin1 (Figure 8E) and the area of PSD-95 clusters (Figure 8F). Pin1 overexpression still exerted a limiting effect on the size of N3A PSD-95 puncta (Figure 8G), suggesting that these effects are independent of binding to the N-terminus domain of PSD-95. These findings support the idea that Pin1 binding to the phosphorylated N-terminus domain of PSD-95 regulates how much PSD-95 is added onto nascent or preexisting PSD clusters. This regulation contributes to a steady-state process of synaptic weakening that is downstream of proline-directed kinases known to phosphorylate this region.

## Discussion

It is known that the PSD is enriched with proteins containing phosphorylated serine/threonine-proline (S/T-P) motifs clustered within a short region of the proteins (Coba et al., 2009). However, only a handful of papers show that some of these are Pin1 targets (Park et al., 2013; Antonelli et al., 2016). This paper tackles this issue by showing a role for Pin1 in the regulation of PSD-95. Specifically, we show how Pin1 binding to the N-terminus PEST domain of PSD-95 regulates the number of functional excitatory synapses. At the biochemical level, Pin1 binds phosphorylated T19 and S25 within the N-terminus domain of PSD-95, and this association strongly decreases the amount of palmitoylated PSD-95. We measured decreases in PSD-95 palmitoylation by Pin1 using two methods, a data set using the click chemistry palmitoylation assay (data not shown) and the hPF11:EGFP intrabody. And with both methods we observed a 70% reduction in total PSD-95 palmitoylation by Pin1. Moreover, the hPF11 results add a new layer to this picture in which it shows the spatial location of palmitoylated PSD-95 in cells. In HEK 293T cells we observed palmitoylated PSD-95 in organelles, which resemble the Golgi apparatus, and in neurons the hPF11 was enriched within dendritic spines with an enrichment ratio of 1.5. The intracellular distribution in HEK 293T cells resembles the signal from intracellular organelles when DHHC2 or DHHC15 are co-expressed with PSD-95 (Fukata et al., 2004; Jeyifous et al., 2016).

The decrease in PSD-95 palmitoylation limits the capacity of PSD-95 into PSDs, ultimately reducing the number of functional synapses. Although Pin1 overexpression produces a strong loss of the number of PSD-95 clusters along the dendritic tree, some clusters and synapses have normal amounts of PSD-95 and post-synaptic AMPARs. We think that the remaining normal synapses may emerge due to multiple scenarios. One scenario is that there is not enough Pin1 to bind all phosphorylated PSD-95, which is very likely given that we overexpressed Pin1 by just a little (∼2-fold). The second scenario is that not all PSD-95 is phosphorylated at T19 and S25 as shown by Morabito et al. (2004). The fact that some synapses lose PSD-95 while others do not suggests that the Pin1-overexpressing neurons experience a normalization mechanism to compensate for the loss of PSD-95 palmitoylation over time, perhaps in the manner described by the findings of Levy et al. (2015) where preexisting palmitoylated PSD-95 is redistributed to its remaining functional synapses. This phenomena is also seen when PSD-95 is knocked down (Béïque et al., 2006; Ehrlich et al., 2007; Sun and Turrigiano, 2011; Levy et al., 2015).

The reduced number of dendritic clusters of PSD-95 was rescued by two manipulations aimed at increasing the total levels of palmitoylated PSD-95 (i.e. Palm B pretreatments and PSD-95 overexpression). In HEK cells, the effects of Palm B were dependent on whether PSD-95 could be phosphorylated, although Pin1 appeared to affect also global palmitoylation. Pin1 strongly limited the Palm B-mediated increase in intracellular clusters in cells expressing wt PSD-95 but not in cells overexpressing the N3A mutant (Figure 1C). These results suggest that Pin1 binds this region to prevent PSD-95 palmitoylation. In neurons, the effects of PSD-95 overexpression were less sensitive to the phosphorylation state of PSD-95 because PSD-95 overexpression of either wt or the N3A mutant.

The Pin1 global knockout and our results show an increase in the size and number of post-synaptic spines. We find that overexpression of Pin1 triggered a loss of functional synapses and a reduction in the diversity of the types of post-synaptic spines. Similarly, Stallings et al., 2018 in found a decrease in spine number when the TAT-WW domain protein was applied to neurons. These results are in line with our findings as we observe spine loss when we overexpressed dominant negative forms of Pin1 (including the WW domain, data not shown). This bidirectional regulation (down or upregulation) of the number of spines by Pin1 suggests that Pin1 is a negative regulator of spine development in hippocampal neurons.

Another interesting aspect of our data is that Pin1 binding, and not isomerization, is sufficient for the effects on PSD-95 palmitoylation. Although Pin1 can isomerize PSD-95, no physiological role was observed for the C-terminal isomerase domain in Pin1. This conclusion may be incomplete because the WW domain of Pin1, over the second time scale, can cause shifts in *cis–trans* equilibrium that are indistinguishable from the conformational changes induced by the isomerase domain (Eichner et al., 2016). This consideration is particularly applicable to this data because the experiments in this work were performed several days after transfecting neurons. These long periods provide enough time for the WW domain to mediate a “quasi isomerization” of the phosphorylated targets that would look indistinguishable from the effects of the isomerase domain. Furthermore, this “quasi isomerization” could be increasingly complicated by the slower turnover rate of PSD-95. Whether or not the isomerase domain is needed for the decrease in PSD-95 palmitoylation is still an open matter for discussion.

Of physiological relevance, Pin1 is strongly upregulated in the striatum and substantia nigra of mice injected with 30mg/kg of MPTP (Ghosh et al., 2013). MPTP administration triggered a six-fold increase in total Pin1 protein levels 12 to 24 h post-stimulation, suggesting that Pin1 protein levels can quickly respond to alterations in neuronal physiology. In our experimental conditions, the observed physiological effects were obtained when Pin1 was overexpressed at levels no more than twice the levels of control cells, suggesting that this mechanism may play a role in striatal physiology as well. In conclusion, this data implicates Pin1 in the regulation of PSD-95 at synapses in normal physiology, synaptic plasticity, or pathological conditions.

## Data Availability Statement

The datasets generated for this study are available on request to the corresponding author.

## Ethics Statement

All procedures were in accordance with guidelines of and approved by the Institutional Animal Care and Use Committee at The University of Chicago.

## Author Contributions

JD conceived the initial idea. JD and DN performed experiments, generated graphs and analyzed data. All authors contributed to all parts of writing and editing the manuscript.

## Conflict of Interest

The authors declare that the research was conducted in the absence of any commercial or financial relationships that could be construed as a potential conflict of interest.
